# The Bcl-2-associated athanogene gene family in tobacco (*Nicotiana tabacum*) and the function of *NtBAG5* in leaf senescence

**DOI:** 10.3389/fpls.2023.1108588

**Published:** 2023-02-09

**Authors:** Linxin Gu, Bing Hou, Xiao Chen, Yu Wang, Pingan Chang, Xiaohong He, Daping Gong, Quan Sun

**Affiliations:** ^1^ Chongqing Key Laboratory of Big Data for Bio Intelligence, College of Bioinformation, Chongqing University of Posts and Telecommunications, Nan'an, Chongqing, China; ^2^ Tobacco Research Institute, Chinese Academy of Agricultural Sciences, Qingdao, China

**Keywords:** tobacco, BAG protein, leaf senescence, *Nicotiana tabacum*, Bcl-2-associated athanogene

## Abstract

Leaf senescence in tobacco is closely related to leaf maturation and secondary metabolites. Bcl-2-associated athanogene (BAG) family members are highly conserved proteins and play key roles in senescence, growth and development, and resistance to biotic and abiotic stresses. Herein, the BAG family of tobacco was identified and characterized. In total, 19 tobacco BAG protein candidate genes were identified and divided into two classes, class I comprising *NtBAG1a–e*, *NtBAG3a–b*, and *NtBAG4a–c* and class II including *NtBAG5a–e*, *NtBAG6a–b*, and *NtBAG7*. Genes in the same subfamily or branch of the phylogenetic tree exhibited similarities in gene structure and the *cis*-element on promoters. RNA-seq and real-time quantitative reverse transcription polymerase chain reaction (qRT-PCR) revealed that the expression of *NtBAG5c–f* and *NtBAG6a–b* was upregulated in senescent leaves, implying that they play a role in regulating leaf senescence. *NtBAG5c* was localized in the nucleus and cell wall as a homology of leaf senescence related gene *AtBAG5*. Further, the interaction of NtBAG5c with heat-shock protein 70 (HSP70) and sHSP20 was demonstrated using yeast two-hybrid experiment. Virus-induced gene silencing indicated that *NtBAG5c* reduced the lignin content and increased superoxide dismutase (SOD) activity and hydrogen peroxide (H_2_O_2_) accumulation. In *NtBAG5c*-silenced plants, the expression of multiple senescence-related genes cysteine proteinase (*NtCP1)*, *SENESCENCE 4* (*SEN4*) and *SENESCENCE-ASSOCIATED GENE 12* (*SAG12*) was downregulated. In conclusion, tobacco BAG protein candidate genes were identified and characterized for the first time.

## Introduction

Leaf senescence is a common process during plant growth and development and is regulated by variety of intrinsic factors, such as cell death, hormones, physiological and biochemical metabolism, senescence-related gene regulation, and environmental factors (Jahan et al., 2021). Leaf senescence is usually accompanied with a visible color change from green to yellow or brown (Koyama, 2018). The internal structure of the leaf undergoes significant changes during senescence, manifested by an abnormal chloroplast structure and decreased in chlorophyll content (Jahan et al., 2021). Decreased of nucleic acid and protein contents and cytoprotective enzyme activity as well as enhanced membrane lipid peroxidation are also observed during plant leaf senescence. The expression of numerous genes, such as senescence-associated genes (SAGs), is upregulated at the onset of senescence, whereas that of photosynthesis- and chlorophyll biosynthesis-related genes is downregulated (Hortensteiner, 2006; Li et al., 2020). Leaf senescence also causes the excessive accumulation of reactive oxygen species (ROS) (Wu et al., 2012; Gutle et al., 2016). In Arabidopsis, ROS are considered as signaling molecules during leaf senescence (Cui et al., 2013).

The Bcl-2-associated athanogene (BAG) protein family acts as a cochaperone that participates in various cellular processes, including stress responses, proliferation, migration, and cell death (Takayama and Reed, 2001; Doong et al., 2002; Kabbage and Dickman, 2008). The first BAG protein, BAG-1, was identified using the protein interacting cloning technique (Takayama et al., 1995). To date, six members of the BAG family have been identified in humans (Song et al., 2001; Takayama and Reed, 2001; Doukhanina et al., 2006; Anderson et al., 2010; Bruchmann et al., 2013). BAG family genes are evolutionarily conserved, with homologs found from yeast to animals. BAG proteins in plants are similar to those in animals and are mainly involved in cell development and apoptotic cell death. However, unlike their homologs in animals, there has been limited study on plant BAG proteins, although the first plant BAG protein was identified in *Arabidopsis* in 2006 (Doukhanina et al., 2006). Subsequently, seven additional members of the *Arabidopsis* BAG family were identified (Fang et al., 2013). Four of these family members (*AtBAG1–4*) encode domain structures outside of the BAG domain (BD) that are similar to those in animal BAGs (Kabbage et al., 2017). AtBAG5–7 is characterized by a tightly distributed calmodulin-binding motif (IQ motif) upstream of the BD. This IQ motif is a specific calmodulin-binding site, which is unique to plant BAG proteins (Kabbage and Dickman, 2008; Kabbage et al., 2016; Kabbage et al., 2017).

The BAG protein family is characterized by the presence of a conserved C-terminal BAG structural domain, which interacts with the ATPase structural domain of heat-shock protein 70 (HSP70/HSC70) to regulate the activity of this chaperone protein (Wang et al., 1996; Takayama et al., 1999; Kabbage and Dickman, 2008). To date, it has been confirmed that BAG proteins can interact with HSP70. In *Arabidopsis*, BAG proteins act as a cofactor in Hsp70-mediated proteasomal degradation of unimported plastid proteins or in plant growth and development (Fang et al., 2013; Lee et al., 2016). AtBAG4 interacts with SNF7.1, NBR1, and Hsp70 and is involved in abiotic stress tolerance (Doukhanina et al., 2006; Arabidopsis Interactome Mapping, Consortium 2011). The CaM/BAG5/Hsc70 complex in *Arabidopsis* regulates leaf senescence (Li et al., 2016a). In addition, AtBAG6 and AtBAG7 may play roles in disease resistance, autophagy, and heat and cold tolerance through the potential interactor C2 GRAM domain protein and an aspartyl protease (Li et al., 2016b), immunoglobulin-binding protein 2 (Williams et al., 2010), WRKY29, and small ubiquitin-like modifier (Li et al., 2017b).

In tobacco, one of the oldest model plants, leaf senescence is associated with the maturation of leaves and rapid formation of secondary metabolites. To date, studies on the senescence in plant leaves have been mainly performed in the model plant *Arabidopsis* and various cash crops, such as rice, wheat, and cotton. However, to the best of our knowledge, there have been no systematic studies on BAG proteins in tobacco, an important protein family with regard to leaf senescence. To bridge this research gap, we identified *BAG* gene family members in tobacco for the first time and analyzed their phylogenetic relationships, gene structures, and chromosomal locations. Moreover, we determined the expression profile of *NtBAG* genes and the protein interactions of candidate NtBAGs. These results obtained here may provide valuable information for exploring the molecular mechanisms of leaf senescence.

## Materials and methods

### Plant materials and growth conditions

The tobacco (*Nicotiana tabacum*) cultivar K326 was used to analyze gene expression patterns. Seeds were sowed into 2:1 vermiculite:turfy soil and cultured at a constant temperature of 22°C under a 16/8 h light/dark cycle in a culture room. All materials were immediately frozen in liquid nitrogen and stored at −70°C until RNA isolation.

### Identification of BAG proteins in tobacco

The genome sequences of *Nicotiana tabacum* (version Nitab4.5) were downloaded from the National Center for Biotechnology Information (NCBI) (https://www.ncbi.nlm.nih.gov/bioproject/?term=PRJNA376174) or Sol Genomics Networ (SGN) (web: https://solgenomics.net/organism/Nicotiana_tabacum/genome and ftp://ftp.sgn.cornell.edu/genomes/Nicotiana_tabacum/edwards_et_al_2017/) (Sierro et al., 2014; Edwards et al., 2017). In addition, the BD sequence was downloaded from Pfam database (accession no. PF02179.17) (Finn et al., 2010) and used as a query for a search in the *Nicotiana tabacum* protein data using HMMER 3.0 with an E-value of<1e^−5^ (Finn et al., 2011). To confirm the results obtained using the HMMER algorithm, the BD sequence was further verified using Pfam and Smart databases (Finn et al., 2010; Letunic et al., 2015; Letunic and Bork, 2018). The BAG protein sequences of *Arabidopsis thaliana* were downloaded from the *Arabidopsis* information resource website (https://www.arabidopsis.org).

### Three-dimensional structural analysis

The structures of NtBAG proteins were predicted using Iterative Threading Assembly Refinement (I-TASSER) server (http://zhanglab.ccmb.med.umich.edu/I-TASSER) (Yang and Zhang, 2015). This server is an online resource for automated prediction of protein structures and structure-based functional annotation. In I-TASSER server, structural templates are first identified from the Protein Data Bank using the multithreaded alignment method, after which full-length structural models are constructed *via* iterative fragment assembly simulations. Considering the confidence level (C-score) of the prediction model, we obtained five simulated structures for each of the provided NtBAG protein sequences. For each sequence, the predicted result with the highest C-score value was selected for further analysis.

### Sequence and phylogenetic analysis

The subcellular localization of proteins was predicted using Plant-mPLoc (http://www.csbio.sjtu.edu.cn/bioinf/plant-multi/#) (Chou and Shen, 2010). We used the ClustalW to perform multiple sequence alignments of BAG proteins obtained from *N. tabacum* and *A. thaliana* (Thompson et al., 1997). A phylogenetic tree was constructed using MEGA 7.0 software and the maximum likelihood method based on the Poisson correction model, with 1000 bootstrap replicates (Tamura et al., 2013). A schematic of the gene structure was constructed using the online software of the GSDS2.0 server (http://http://gsds.gao-lab.org/index.php) (Hu et al., 2015). Data regarding the chromosomal location of *NtBAG* were obtained from the genome of *N. tabacum*. We subsequently mapped these *BAG* genes using MapInspect software. Conserved protein motifs were identified using default parameters for Multiple Em for Motif Elicitation (http://meme-suite.org/) program, with a maximum of 12 motifs. The subcellular localization of NtBAGs was predicted using ProtComp9.0 (www.softberry.com), and the identified protein motifs were further annotated using Weblogo (http://weblogo.berkeley.edu/). Finally, a 1500-bp segment of the 5′ sequence was used as the promoter region of each BAG gene to analyze the *cis*-acting elements using PlantCARE (http://bioinformatics.psb.ugent.be/webtools/plantcare/html/) (Lescot et al., 2002).

### Expression profile of *BAG* genes based on RNA sequencing data

RNA sequencing (RNA-seq) data were downloaded from the NCBI Sequence Read Archive database (http://www.ncbi.nlm.nih.gov/sra/) with the accession numbers SRP029183 and SRP029184. Clean reads filtered from raw reads were mapped onto the *N. tabacum* genome using HISAT (version Nitab4.5) with the default parameters (Kim et al., 2015). The levels of expression of individual genes were quantified in terms of transcripts per kilobase of exon model per million mapped read (TPM) values, which were obtained using StringTie with the default parameters (Pertea et al., 2015; Pertea et al., 2016).

### Real-time quantitative reverse transcription polymerase chain reaction analysis

Total RNA was extracted from different plant materials using RNA Plant Kit (Takara, Qingdao, China) and treated with DNase I (Takara, Qindao, China) to remove genomic DNA. Reverse transcription was performed using the HiScript II 1^st^ strand complementary DNA (cDNA) synthesis kit (Vazyme, Nanjing, China). qRT-PCR was performed using a 20 μL reaction volume comprising 10 μL SYBR qPCR Master Mix (Vazyme), 6.4 μL of ddH_2_O, 0.8 μL of forward primer (10 μmol/L), 0.8 μL of reverse primer, and 2 μL of template cDNA. Nt36s was used as the internal reference gene for qRT-PCR. The gene-specific primers are listed in **Table S1**.

Three replicates corresponding to each period were subjected to amplification using Bio-Rad IQ5 Real-Time PCR instrument (Bio-Rad Laboratories, Hercules, CA, USA). The amplification parameters were as follows: activation at 50°C for 2 min, predenaturation at 95°C for 2 min, denaturation at 95°C for 15 s, and annealing at 60°C for 1 min (40 cycles). Finally, the relative gene expression was calculated using the 2^−ΔΔCt^ method (Livak and Schmittgen, 2001).

### Subcellular localization of *NtBAG5c* protein

*NtBAG5c* was cloned into the PCAMBIA1300-35S-GFP vector and transformed into *Agrobacterium tumefaciens* strain LBA4404. The primers were designed based on *NtBAG5c* sequence (**Table S1**). *Agrobacterium* containing the control GFP vector or NtBAG5c-GFP recombinant vector was injected into the abaxial side of the leaves (2–4 weeks old). The leaves were then incubated in the dark for 2–3 days. Subsequently, 4′,6-diamidino-2-phenylindole (DAPI) (Solarbio, Beijing, China) was used for nuclear counterstaining. Approximately 5 min after staining, the tissue was washed twice or thrice with phosphate-buffered saline. The plasmolysis experiments were identified according to methods previously reported (Zhang et al., 2022). Finally, the tissue was sealed with a cover slip and examined under a microscope (IX73, OLYMPUS, Japan).

### Yeast two-hybrid experiment

Total leaf RNA was extracted from *N. benthamiana* and reverse transcribed to obtain cDNA. The coding sequences of the *NtBAG5c* and *HSP70* genes were amplified *via* PCR using the designed primers (**Table S1**), which were cloned into the *Eco*R I and *Bam*H I sites of the pGBKT7 and pGADT7 vectors, respectively. The yeast receptor cells transformed with plasmid combinations pGBKT7-NtBAG5c + pGADT7-HSP70, pGADT7 + pGBKT7-NtBAG5c, pGBKT7 + pGADT7-HSP70, pGADT7-T + pGBKT7-53, pGADT7-T + pGBKT7-Lam, and pGADT7 + pGBKT7 were inoculated onto two-deficiency synthetic defined (SD) medium and incubated upside-down at 30°C for 2–3 days. Then, colonies with a diameter of >2 mm were transferred to a four-deficiency SD medium and incubated upside-down at 30°C for 4–5 days.

### Virus-induced gene silencing of *NtBAG5c*

To explore the role of *NtBAG5c* gene in leaf senescence, tobacco rattle virus (TRV)::NtBAG5c vector was firstly constructed and empty vector (TRV::00) was used as a control. The tobacco phytoene desaturase (PDS) fragments were cloned into TRV vectors to construct TRV::PDS, which was used as a positive control. The constructed cloning vector was transformed into *Agrobacterium tumefaciens* GV3101. The primers used in this experiment are shown in **Table S1**. Virus-induced gene silencing (VIGS) was performed as described previously (Bachan and Dinesh-Kumar, 2012). Then, a buffer containing pTRV1 was mixed with TRV::00, TRV::PDS, or TRV::NtBAG5c at a ratio of 1:1 by volume. The plants in which the fourth leaf had fully expanded were used for VIGS. Small holes were punched with a needle on the underside of the leaves to facilitate infiltration. The inoculated plants were grown at 20°C for 24 h under relative humidity of 60% in the dark, and then placed in a growth room at 25°C with a 16-h light/8-h dark photoperiod. Ten days later, the leaves were obtained for subsequent experiments. The assays were performed with at least ten plants for each vector, and the experiments were repeated at least three times.

### Determination of chlorophyll content

On day 10 after of VIGS, the third functional leaf (0.2 g, from the top to bottom) was sliced into pieces and 20 mL of acetone:ethanol:water (4.5:4.5:1 [v:v]) was added to the leaves; the mixture were stored in the dark. The OD_645_ and OD_663_ values were measured after the leaves turned white. The chlorophyll content was calculated using the following formulas (He et al., 2021):


$$Chl\;a(mg/g)=(12.7\times OD_{663}-2.69\times OD_{645})\times V(mL)/[1000\times m(g)]$$

$$Chl\;b(mg/g)=(22.9\times OD_{645}–4.68\times OD_{663})\times V(mL)/[1000\times m(g)]$$

### Determination of lignin content

Lignin content was measured using ultraviolet spectrophotometry in accordance with the manufacturer’s instructions for the kit (BC4200; Beijing Solarbio Science Technology Co., Ltd., Beijing, China) (Liang et al., 2020).

### Superoxide dismutase activity assay

The total SOD activity was determined using a kit (Nanjing Jiancheng Bioengineering Institute, Nanjing, China). One unit of SOD activity is defined as the amount of enzyme required for 1 mg of tissue proteins in 1 ml reaction volume to achieve a SOD inhibition rate of 50%, as monitored at 550 nm. SOD activity is presented as U mg^−1^ protein.

### Analysis of hydrogen peroxidase accumulation

H_2_O_2_ accumulation was detected using 3,3′-diaminobenzidine (DAB) staining. The fifth and sixth leaves of negative control plants (pTRV2:00) and NtBAG5c-silenced plants were immersed overnight in DAB solution (1 mg/mL; pH 3.8) overnight to detect the *in-situ* accumulation of H_2_O_2_. Leaves were fixed with 100% ethanol for 3 h and then boiled in 95% ethanol for 10 min to remove chlorophyll before imaging (Li et al., 2016a).

### Statistical analyses

Standard error of mean was calculated using GraphPad Prism, and statistical significance was determined using paired *t*-test *via* Excel. All data are presented as the mean (± SEM) of three independent biological determinations.

## Results

### Identification of BAG family members in *N. tabacum*

To identify BAG proteins in *N. tabacum*, we screened out 19 genes and confirmed the domains using Pfam and Smart databases. On the basis of similarity with homologous genes in *A. thaliana*, the 19 *NtBAG* genes were named *NtBAG1a*–*NtBAG7* (**Table S2**). The coding proteins range from 196 to 1273 amino acids (aa), with a molecular weight of 22.4–141.76 kDa and an isoelectric point (pI) of 4.59–9.56. We also predicted that most NtBAG were localized in the nucleus, while NtBAG3a, NtBAG5c, and NtBAG7 were also localized in chloroplasts and NtBAG3a was localized in mitochondria (**Table S2**). Of the 19 BAG genes, 9 were located on 7 chromosomes, whereas the other genes were anchored in multiple scaffolds.

### Phylogenetic tree of *NtBAG*s and *AtBAG*s

**Figure 1:**
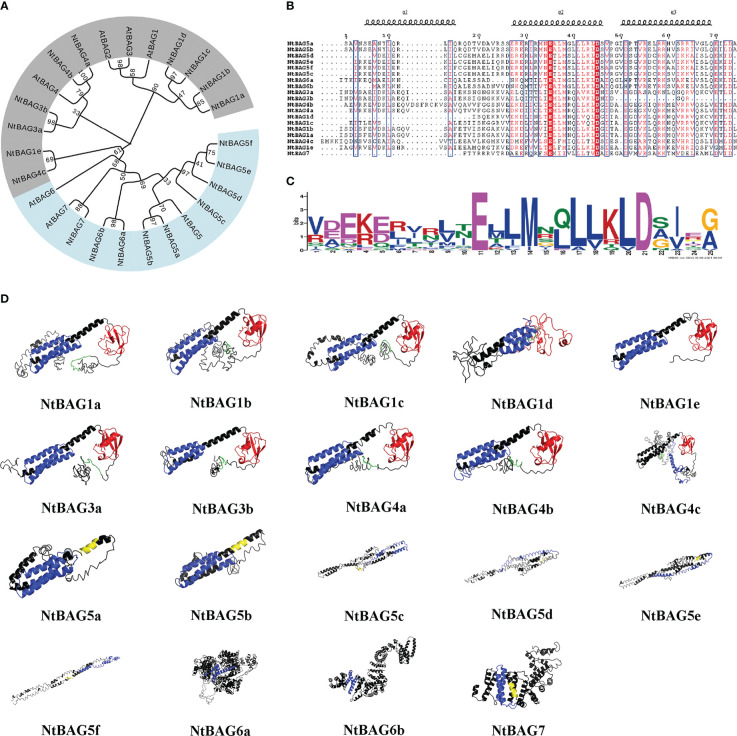
Phylogenetic and three-dimensional structure analysis of the NtBAG proteins. **(A)** The evolutionary history was inferred using the maximum likelihood method based on the Poisson correction model. The bootstrap consensus tree inferred from 1,000 replicates is taken to represent the evolutionary history of the analyzed taxa. Branches corresponding to partitions reproduced in less than 50% of bootstrap replicates are collapsed. Initial trees for the heuristic search were obtained automatically by applying neighbor-joining and BioNJ algorithms to a matrix of pairwise distances estimated using a JTT model, and then selecting the topology with the superior log likelihood value. The analysis involved 86 amino acid sequences. All positions containing gaps and missing data were eliminated. There were a total of 66 positions in the final dataset. Evolutionary analyses were conducted in MEGA7. **(B)** Alignment of the domains conserved within the BAG protein family. The locations of the three helices are shown above the alignment. **(C)** A motif conserved among the BAG family proteins. The consensus sequences were displayed using Weblogo. **(D)** Prediction of three-dimensional structure of NtBAG proteins. The BDs of the NtBAG proteins are depicted in blue, the ubiquitin domain (UBD) is depicted in red, and the IQ motif is depicted in yellow. A conserved sequence motif of 12 amino acids (ExRPGG[ML/VV]QxR) is depicted in green.

To assess the phylogenetic relationships among the members of the BAG family, we used the predicted BAG protein sequences from *N. tabacum* and *A. thaliana* to construct a phylogenetic tree. The BAG proteins could be divided into two groups: class I (gray) and class II (blue) (**Figure 1A**). The class I BAG proteins mainly comprised AtBAG1 to AtBAG4, clustered with AtBAG1/AtBAG2 homologous proteins NtBAG1a–e and NtBAG3a–b as well as AtBAG4 homologous proteins NtBAG4a–c. The class II BAG proteins mainly included AtBAG5–7 clustered with their homologous proteins, NtBAG5a–e, NtBAG6a–b, and NtBAG7 (**Figure 1A**).

NtBAGs typically have three helices in the conserved domain and often possess Glu and Asp residues at positions 11 and 21 of the conserved domain, respectively (**Figures 1B, C**).

### Three-dimensional structural analysis

Structural analysis of NtBAGs was performed based on predicted structures obtained using I-TASSER server. The predicted structure with the highest C-score was selected as a representative structure for further analysis (**Figure 1D**). The structures of NtBAG1a, NtBAG1b, NtBAG1c, NtBAG1d, NtBAG1e, NtBAG3a, NtBAG3b, NtBAG4a, NtBAG4b, and NtBAG4c were comparable in terms of the presence and organization of the major structural domains. Their structures contained a highly organized BD and an ubiquitin-like domain. The conserved sequence pattern of the characteristic 12 aa in the N-terminal domain, which is found in all family members except NtBAG1e, showed a hairpin loop pattern. The highly conserved ubiquitin-like structural domain strictly exhibited four β-sheets and two α-sheets. Of the four β-sheets, two were organized as a central β-sheet with an α-helix on either side. The BAG structural domain of all NtBAGs consists of a typical three α-helix bundle structure. The IQ-calmodulin binding pattern in NtBAG5c is a small α-helix connected by two hairpin loops, whereas that in NtBAG5a, NtBAG5b and NtBAG7 is a complete single helix.

### Structures and conserved motif analysis of *NtBAGs*

**Figure 2:**
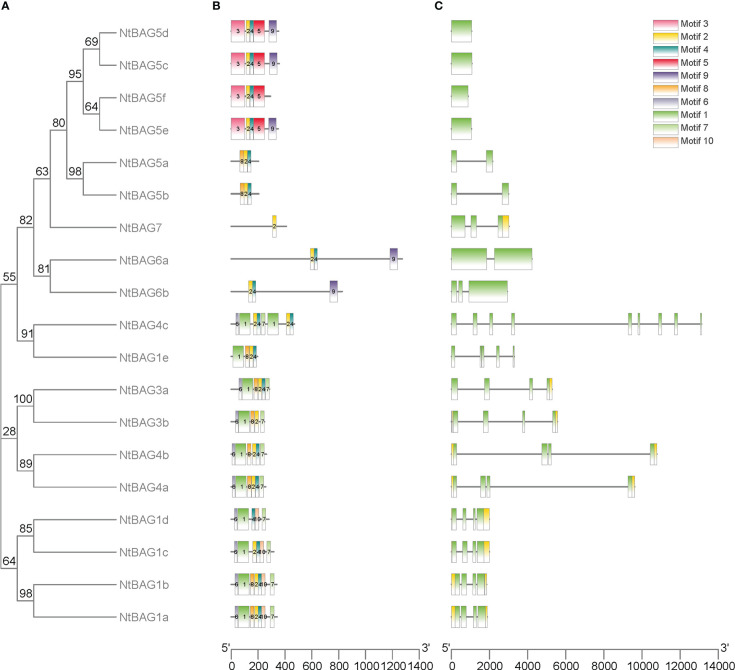
Genomic structure and motif composition of NtBAGs. **(A)** The phylogenetic tree of NtBAG proteins. **(B)** The conserved motifs in NtBAG proteins were identified using MEME. Each motif is represented by a specific color and the nucleotide sequence numbers are shown below. **(C)** Genomic structure of NtBAG family members in tobacco. Exons and introns are represented by green boxes and black lines, while yellow box shows the noncoding region.

To further analyze the characteristics of *NtBAG*s, we explored the conserved motifs and gene structure in terms of exons and introns. The conserved motifs in these genes also showed similarities within the same subgroup, such as six similar motifs in all NtBAG1–4 homologous proteins, except for NtBAG1e and NtBAG4c (**Figure 2A, B**). NtBAG6a–b had three motifs, whereas NtBAG7 only had one of the ten motifs. Among the six NtBAG5 homologous proteins, NtBAG5a–b had three motifs, whereas NtBAG5c–e had four to five motifs. Gene structure analysis indicated that most *NtBAG1–4* homologous genes had four exons, except for *NtBAG1e* and *NtBAG4c*, which contained five and nine exons, respectively. NtBAG5a–b, NtBAG5c–f, and NtBAG6–7 contained two, one, and two to three exons, respectively. Further, we revealed that the genetic structures of all BAG family proteins from *N. tabacum* were similar, with them showing close evolutionary relationships. Moreover, genes within the same subfamily often showed similar gene structures (**Figures 2A, C**).

### Promoter *cis*-acting element analysis of *NtBAGs*

The *cis*-acting elements in the promoter usually regulate gene expression and function. Multiple *cis*-acting elements, such as plant hormone, light, and stress response elements, were identified in BAG gene promoters (**Figure S1** and **Table S3**).

Regarding hormone-related *cis*-acting elements, we identified at least one abscisic acid response element (ABREs) in the promoters of *N. tabacum* BAG genes, except for *NtBAG1e*, *NtBAG5b–c*, and *NtBAG5e–f*. Further, only one auxin response element (TGA element) was detected in the promoters of *NtBAG3a*, *NtBAG4b*, *NtBAG5d*, and *NtBAG7* promoters. Meanwhile, at least one of the gibberellin response elements, such as TARC-box, P-box, or GARE, was found in the promoters of most genes, except for *NtBAG1a–b*, *NtBAG4a, NtBAG4c*, *NtBAG5c–e*, and *NtBAG6a*. The Methyl Jasmonate (MeJA) response elements CGTCA and TGACG, were identified in the promoters of most genes, except for *NtBAG4a*, *NtBAG4c*, *NtBAG5a*, and *NtBAG6a*. Further, we found several other hormone-related *cis*-elements, such as the salicylic acid (SA) response element TCA element, in some *NtBAG* promoters.

In addition, we identified numerous *cis*-acting elements related to light response in these promoters, including ACE, AE box, TCT motif, ATC motif, Box 4, GATA motif, G Box, and GT1 motif. Among them, G box is distributed in the promoters of most members of the *NtBAG* family. We also detected stress response-related *cis*-acting elements in these promoters, including LTR, MBS, and TC-rich repeats, along with other elements, such as WUN motif (related to wounds); circadian element (related to circadian control); and Myb, Myc, STRE, TC-rich, W box, and ARE elements. In particular, we identified Myb and Myc-motif elements in almost all BAG promoters.

### Tissue-specific expression profiles of *NtBAG*s

**Figure 3:**
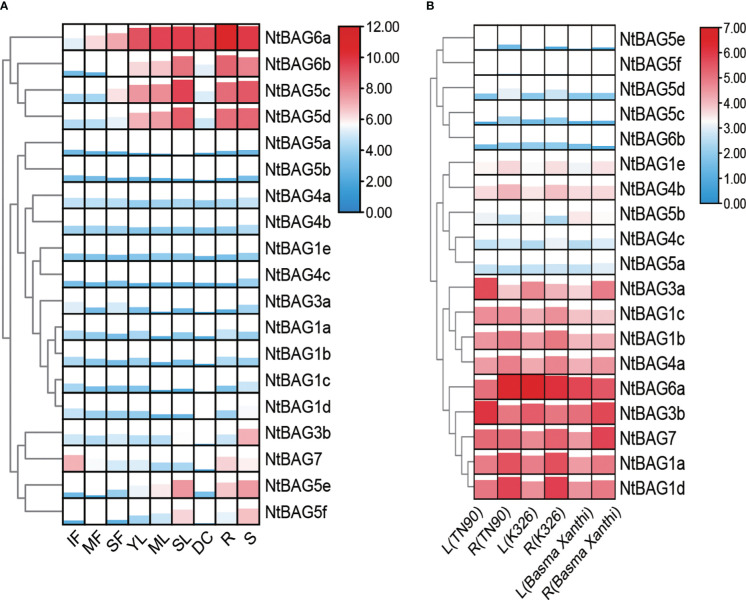
Expression patterns of *BAG* genes. **(A)** Expression patterns in different tissues and developmental stages of tobacco. **(B)** *BAG* gene expression patterns of leaf and root in different varieties. MF, mature flower; R, root; DC, dry capsule; ML, mature leaf; SF, senescent flower; SL, senescent leaf; S, stem; IF, immature flower; YL, young leaf. The expression levels are represented by the color bar (log2-transformed).

Based on the previously obtained RNA-seq data (Sierro et al., 2014), the expression patterns of all *BAGs* in different developmental periods and tissues were analyzed. *BAG*s were divided into four clusters according to their expression trends. The first cluster contained NtBAG5c–d and NtBAG6a–b, which were highly expressed in senescent flower (SF), young leaf (YL), mature leaf (ML), senescent leaf (SL), root (R), stem (S), dry capsule (DC), and other tissues. Moreover, the expression patterns of these genes tended to increase with the aging of flowers and leaf tissues. The second cluster comprised NtBAG1a–e, NtBAG3a, NtBAG4a–b, and NtBAG5a–b, which showed low expression levels in all tissues. NtBAG3b and NtBAG7 constituted the third cluster and showed high expression in R and S tissues. In addition, NtBAG7 was highly expressed in immature flower (IF) tissues, whereas it showed low expression in leaf and flower tissues with aging. The fourth cluster included NtBAG5e–f, which showed a lower expression trend similar to that of NtBAG5c-d (**Figure 3A**). Further analysis revealed a similar in the expression pattern in both root and stem tissues in different varieties (**Figure 3B**).

### Expression of *NtBAG*s during leaf senescence

**Figure 4:**
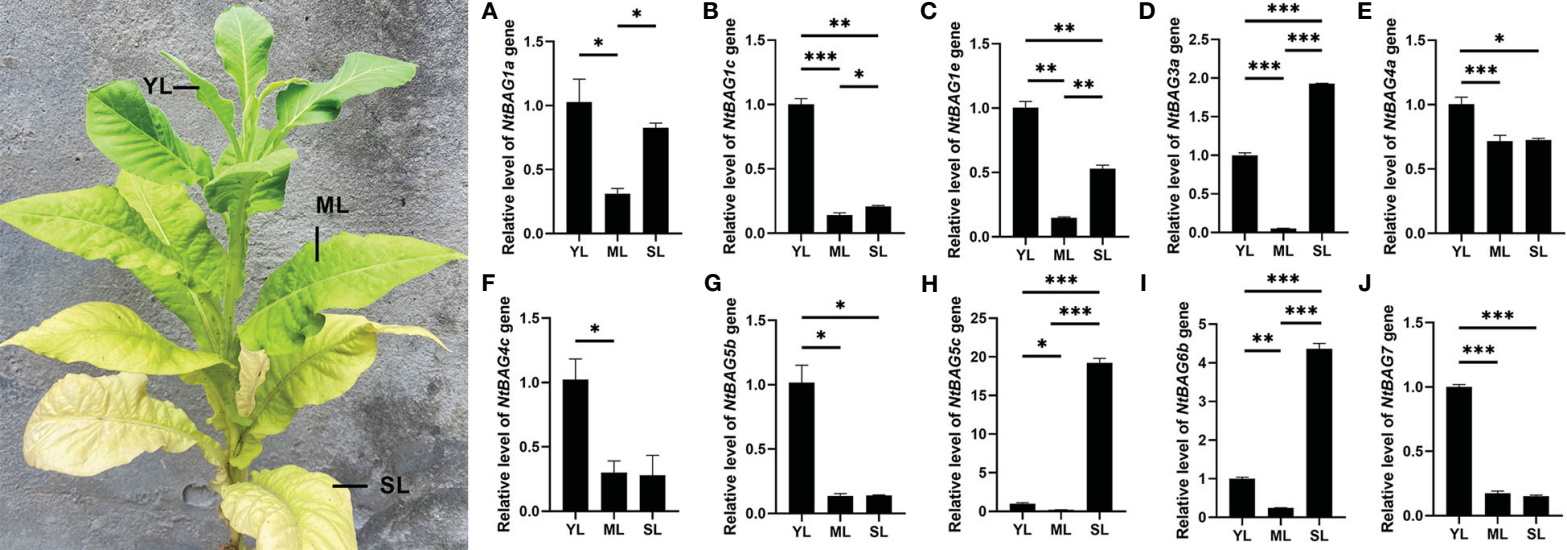
The relative expression pattern of *NtBAG* in wild type tobacco. The expression levels of NtBAG1a **(A)**, NtBAG1c **(B)**, NtBAG1e **C)**, NtBAG3a **(D)**, NtBAG4a **(E)**, NtBAG4c **(F)**, NtBAG5b **(G)**, NtBAG5c **(H)**, NtBAG6b **(I)** and NtBAG7 **(J)** in plant leaves at different developmental periods, as determined by qRT-PCR. YL, young leaf; ML, mature leaf; SL, senescent leaf. All data are presented as the mean (± SEM) of three independent biological determinations and were analyzed by Student’s *t*-test (*p< 0.05, **p< 0.01, ***p< 0.001).

Based on the abovementioned results, multiple *NtBAGs* may be associated with aging. qRT-PCR was used to further identify the genes related to leaf senescence. Compared with young leaves, the expression of *NtBAG1a* was significantly downregulated in mature leaves but was upregulated in senescent leaves (**Figure 4A**). Meanwhile, the expression levels of *NtBAG1c* and *NtBAG1e* were significantly decreased compared with that in young leaves (**Figures 4B, C**). Compared with young leaves, the expression level of *NtBAG3a* was significantly decreased in mature leaves but significantly increased in senescent leaves (**Figure 4D**). The expression patterns of *NtBAG4a*, *NtBAG4c*, and *NtBAG5b* were also highly similar to that of NtBAG1c (**Figures 4E–G**). The expression of *NtBAG5c* and *NtBAG6b* was significantly decreased in mature leaves but was significantly increased in senescent leaves, consistent with the expression of *NtBAG3a* (**Figures 4H, I**). Interestingly, the expression of NtBAG5c showed a sharp variation as it was upregulated in senescent leaves compared with young leaves by approximately 20 times, which was much higher than the expression of *NtBAG3a* and *NtBAG6b* (**Figures 4D, H, I**). The expression of *NtBAG7* was high in young leaves and significantly low in mature and senescent leaves (**Figure 4J**). The expression trends of these genes were consistent with the previous RNA-seq data.

### NtBAG5c localizes in the cell wall and interacts with HSP proteins

**Figure 5:**
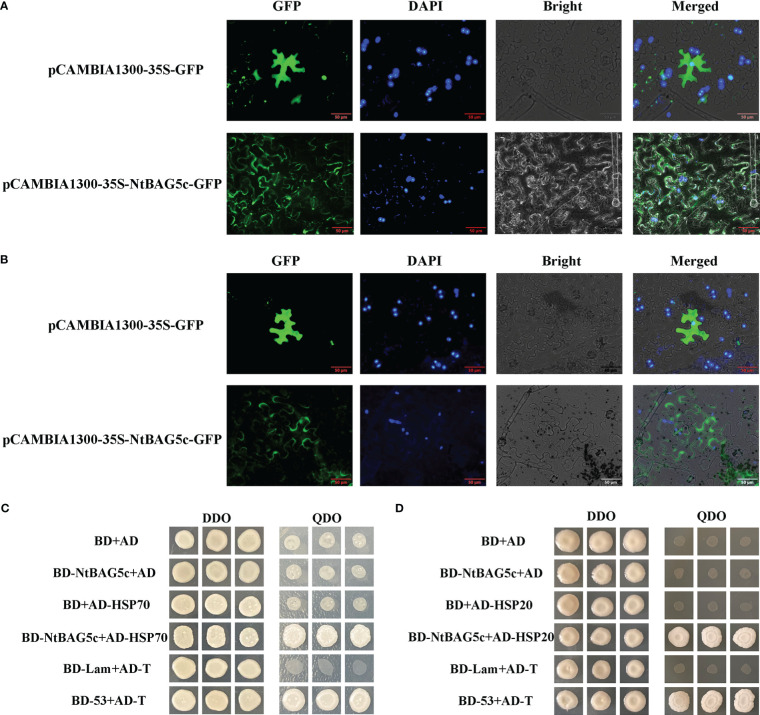
Localization of *NtBAG5c* in epidermal cells of *N. benthamiana*. **(A)** Subcellular localization analysis demonstrated that NtBAG5c is located in the cell membrane and cell wall. **(B)** After the wall separation, subcellular localization analysis indicated that NtBAG5c is located in the cell wall. GFP, green fluorescent protein; DAPI, fluorescent dye capable of binding strongly to DNA; Bright, white light; Merged, superposition of GFP, DAPI, and Bright. **(C, D)** Yeast two-hybrid assay. **(C)** The interaction of NtBAG5c and HSP70 in yeast cells. **(D)** The interaction of NtBAG5c and HSP20 in yeast cells. BD-53 + AD-T and BD + AD were used as positive and negative controls, respectively. The yeast co-transformed groups were grown on the SD Leu-Trp medium [double dropout (DDO), without leucine and tryptophan], and then grown on SD-Leu-Trp-His-Ade medium [quadruple dropout (QDO), with leucine, tryptophan, histidine, and adenine].

Localization prediction using Plant-mPLoc revealed that NtBAG5c was localized in the nucleus and chloroplast. NtBAG5c-GFP fusion protein was observed to be accumulated in the nucleus, cell membrane, and cell wall (**Figure 5A**). After separation of the cell wall, NtBAG5c-GFP fusion protein was localized in the cell wall and nucleus (**Figure 5B**). The nucleus targeting of NtBAG5c-GFP was consistent with the previous prediction (**Figures 5A, B**).

Most members of the BAG family can interact with HSPs (Doong et al., 2000; Alberti et al., 2003; Chung and Dawson, 2004; Davidson et al., 2016). We analyzed the interaction of NtBAG5c proteins with the candidate proteins HSP70 and HSP20 using yeast two-hybrid experiment. The results showed that NtBAG5c can interact with HSP70 and HSP20 *in vitro* (**Figures 5C, D**).

### *NtBAG5c* is involved in leaf senescence

**Figure 6:**
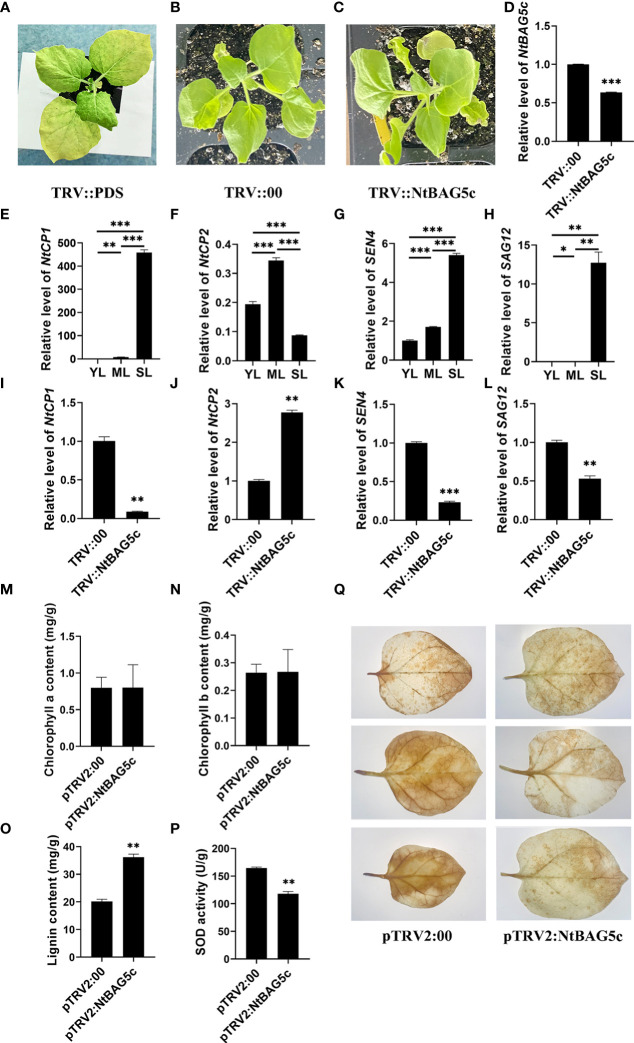
Silencing of NtBAG5c in *Nicotiana tabacum* using a tobacco rattle virus (TRV)-based VIGS system. **(A–C)** Phenotypes of tobacco plants after 10 days of VIGS. **(A)** PDS control plants (*TRV::PDS*); **(B)** negative control plants (*TRV::00*); **(C)** NtBAG5c-silenced plants (*TRV::NtBAG5c*). The expression levels of NtBAG5c **(D)**, NtCP1 **(E)**, NtCP2 **(F)**, SEN4 **(G)**, and SAG12 **(H)** in plant leaves at different developmental periods, as determined by qRT-PCR. The expression levels of NtCP1 **(I)**, NtCP2 **(J)**, SEN4 **(K)**, and SAG12 **(L)** in NtBAG5c-silenced plants by qRT-PCR. **(M)** Chlorophyll a content. **(N)** Chlorophyll b content. **(O)** Lignin content (mg/g). **(P)** SOD activity (U/g). **(Q)** DAB staining of isolated leaves of pTRV2:00 and pTRV2:NtBAG5c. Results were reproduced in three independent experiments using three plants each. All data are presented as the mean (± SEM) of three independent biological determinations and were analyzed using Student’s t-test (*p < 0.05, **p <  0.01, ***p <  0.001).

*NtBAG5c* was silenced in *N. tabacum* using a TRV-based VIGS method. *NtBAG5c-*silenced plants showed no significant phenotypic differences compared with the control plants (**Figures 6A–C**). qRT-PCR analysis revealed that the expression of the *NtBAG5c* in *NtBAG5c*-silenced plants was significantly downregulated compared with that in the negative control (**Figure 6D**), thus confirming the successful silencing of *NtBAG5c*.

The expression of SAGs, such as *NtCP1*, *NtCP2*, *SAG12*, and *SEN4*, was analyzed using further tests. In senescent leaves, the expression levels of *NtCP1*, *SAG12*, and *SEN4* were significantly high and that of *NtCP2* was low (**Figures 6E–H**). These results were consistent with those in previous reports (Nam, 1997; Beyene et al., 2006; Jiang et al., 2014). In *NtBAG5c-*silenced plants, the expression of *NtCP1*, *SEN4*, and *SAG12* was significantly decreased (**Figures 6I, K, L**) but that of *NtCP2* was upregulated (**Figure 6J**). Thus, *NtBAG5c* may act as an upstream regulator to participate in leaf senescence. Although there were evident changes in the expression levels of senescence-related genes, no significant differences in phenotypes were observed between the *NtBAG5c*-silenced and the control plants (**Figures 6A–C**). This was further confirmed by the chlorophyll contents in the leaves of *NtBAG5c*-silenced plants and control plants. Thus, the silencing of *NtBAG5c* did not affect the chlorophyll content in plants (**Figures 6M, N**).

### *NtBAG5c* inhibits lignin formation and promotes H_2_O_2_ generation

The lignin content is lower in senescent leaves of maize mutant is lower than that in the wild type maize (Jiao et al., 2019). Compared with control plants, a significant increase in lignin content was observed in the leaves of *NtBAG5c*-silenced plants (**Figure 6O**), suggesting that *NtBAG5c* plays a role in lignin synthesis.

SOD activity is closely related to leaf senescence and regulates the level of H_2_O_2_ accumulation (Cui et al., 2013; Wang et al., 2016). The SOD activity in *NtBAG5c*-silenced plants was significantly lower than that in control plants (**Figure 6P**), implying that NtBAG5c promotes SOD enzyme activity. Further assays of H_2_O_2_ levels *via* DAB staining revealed that the *NtBAG5c*-silenced leaves were more weakly stained than the control leaves, indicating that NtBAG5c can promote H_2_O_2_ accumulation (**Figure 6Q**).

## Discussion

The formation and composition of secondary metabolites in tobacco leaves during post-maturation can affect the quality of the leaves. Leaf senescence is associated with the maturation of leaves and the rapid formation of secondary metabolites. *BAGs* are involved in plant growth, development, and stress response (Thanthrige et al., 2020). In plants, BAG1 was the first identified member of the BAG family and was shown to interact with BCL-2 by enhancing its antiapoptotic function (Takayama and Reed, 2001). Homologs of the BAG family have been identified in rice (Rashid Mehmood, 2012), tomato (Jiang et al., 2022), wheat (Ge et al., 2016), banana (Dash and Ghag, 2022), and *Arabidopsis* (Yan et al., 2003; Doukhanina et al., 2006; Nawkar et al., 2017). Seven BAG genes were identified in *Arabidopsis*, but we identified 19 homologs in common tobacco. Among these genes, six homologs of AtBAG5 may be the main reason for the increased number of BAG genes. In *Arabidopsis*, the function of AtBAG5 is mainly associated with leaf senescence (Cui et al., 2015; Li et al., 2016a). It remains to unclear whether the direction of selection by scientists during breeding leads to an increase in the number of BAG5 homologs.

Phylogenetic analysis confirmed that BAGs are highly conserved throughout the plant kingdom. The 19 NtBAGs were divided into two groups. The first group comprised *NtBAG1a–e*, *NtBAG3a–b*, and *NtBAG4a–c* clustered with *AtBAG1–4*, whereas the second group comprised *NtBAG5a–e*, *NtBAG6a–b*, and *NtBAG7* clustered with *AtBAG5–7*. Moreover, the structure and conserved motifs of *NtBAGs* showed similarities within the same subgroup. These results imply that *BAGs* in tobacco have highly similar functions to those of the homologous genes in *Arabidopsis*.

*Cis*-acting elements regulate gene transcription. We identified a series of abiotic stress- and hormone-related elements in most *NtBAG* promoters, such as the phytohormone response elements ABRE, CGTCA, and TGACG. Meanwhile, NtBAG1a–e, NtBAG3a–b, NtBAG4a–c, and NtBAG5a had two or more G Box *cis*-acting elements, and transcriptomic data revealed that the expression of these genes was relatively stable over multiple periods and tissues, showing a slight variation compared with that of other genes. The characteristics of continuous and constant expression of these genes are also highly similar to those of AtBAG1–3 in *Arabidopsis* (Vivancos et al., 2010). NtBAG5b–f and NtBAG6a–b have less than one G box *cis*-acting element, and their expression varies dramatically during maturation and senescence. However, it remains unclear whether this element is related to the regulation of senescence during maturation. In addition, stress response-related *cis*-acting elements, including LTR, MBS, and TC-rich repeat sequences, were detected in the promoters of multiple *NtBAGs*, except for NtBAG3b, NtBAG4a, NtBAG4c, and NtBAG5c, suggesting that multiple *NtBAG*s are involved in the plant stress response. This finding is consistent with that of previous studies on *Arabidopsis BAGs*, which also contain stress response elements, including ABRE, ERE, CGTCA motifs, MBS, and TC-rich repeat sequences (Doukhanina et al., 2006; Nawkar et al., 2017). This suggests that *BAGs* play an important role in the resistance of plants to stress.

Based on the expression patterns obtained using RNA-seq data and slight differences in gene sequences within the same cluster in the phylogenetic tree, 10 *NtBAGs* were screened for qRT–PCR analysis. These genes were divided into two categories based on their expression patterns. The first category included genes with high expression in young leaves but significantly low expression in mature and senescent leaves, namely, *NtBAG1a*, *NtBAG1c*, *NtBAG1e*, *NtBAG4a*, *NtBAG4c*, *NtBAG5b*, and *NtBAG7*. The second category comprised genes that showed a significantly higher expression in senescent leaves than in young and mature leaves, namely, *NtBAG3a*, *NtBAG5c*, and *NtBAG6b*. In *Arabidopsis*, BAGs can also be divided into two broad groups based on the organization of their domains. Notably, the expression of NtBAG5c increased dramatically in senescent leaves, which is similar to the expression trend of marker genes of senescence in plants, namely, *NtCP1*, *SEN4*, and *SAG12* (Park et al., 1998; Grbić, 2002; Beyene et al., 2006).

However, regarding the localization of BAGs of *Arabidopsis*, BAG5 was accumulated in mitochondria (Li et al., 2016a), whereas NtBAG5c was mainly localized in the nucleus and cell wall, which may be related to the functional differentiation of multiple BAG5 homologs in tobacco.

The BAG family proteins of *Arabidopsis*, rice (Zhou et al., 2021), and other plants can act as a backbone to link Hsp70 and small heat stress proteins, such as sHsp20, *via* protein interactions, as determined using yeast two-hybrid and immunoprecipitation experiments. The IQ motif in the AtBAG5 can bind to calcium-regulated proteins and thus participate in the regulation of the calcium signaling pathway, forming a complex with Hsp70 and regulating leaf senescence (Li et al., 2016a). Furthermore, the results of yeast two-hybrid experiment showed that NtBAG5c can interact with Hsp70 and Hsp20 *in vitro*, which may form a complex involved in the regulation of leaf senescence in tobacco.

In *Arabidopsis*, BAG5 regulated the production of ROS and expression of SAGs (Li et al., 2016a). H_2_O_2_ can induce ROS production and accelerate leaf senescence. A previous study revealed that NtBAG5c-silenced plants exhibit low levels of H_2_O_2_, which is consistent with the findings of a study on AtBAG5 (Li et al., 2016a). This indicates that NtBAG5c can promote H_2_O_2_ accumulation and accelerate leaf senescence. Moreover, we revealed that NtBAG5c-silenced plants exhibit lower SOD activity and higher lignin content than control plants, suggesting that NtBAG5 can inhibit lignin accumulation and promote SOD activity. Further, a study reported that CsMYB4a overexpressed in tobacco plants can promote senescence and reduce total lignin content (Li et al., 2017a). In tobacco, NtBAG5c may regulate lignin content through its homology with CsMYB4a. In particular, *CP1*, *SEN4*, and *SAG12* are often highly expressed in senescent leaves (Park et al., 1998; Grbić, 2002; Grbić, 2003; Beyene et al., 2006). The expression of *NtCP1*, *SEN4*, and *SAG12* was significantly downregulated in *NtBAG5c*-silenced plants, indicating that NtBAG5c acts as a positive regulator and is involved in the regulation of leaf senescence.

## Conclusions

This study identified and screened 19 *BAG* family members from the tobacco genome. The gene structure, structural domains, physicochemical properties, and expression patterns of tobacco *BAGs* were characterized. Further analysis of the expression of these *NtBAGs* using qRT–PCR revealed that multiple *NtBAGs* may be related to tobacco leaf senescence. Among them, NtBAG5c interacts with Hsp70 and Hsp20, indicating that NtBAG5c affects tobacco leaf senescence by forming a complex with Hsp70 or Hsp20. This study provides a theoretical basis for the further investigation of the *NtBAG* gene family and provides important guidance for molecular breeding.

## Data availability statement

The original contributions presented in the study are included in the article/**Supplementary Material**. Further inquiries can be directed to the corresponding authors.

## Ethics statement

The authors declare that all methods were carried out in accordance with relevant guidelines and regulations.

## Author contributions

QS and DG designed the study. QS carried out bioinformation analyses. LG, BH, YW carried out the qRT-PCR analyses. BH, YW, XH and PC collected plant materials. LG, BH and XC carried out other experiments. LG wrote the original draft. All authors contributed to the article and approved the submitted version.

## Supplementary material

Supplementary Figure 1
*Cis*-acting elements on promoters (1500 bp) of *NtBAG* genes. The number shows the number of *cis*-acting elements.

Supplementary Table 1 Information of primer sequences

Supplementary Table 2 BAG protein family members in *Nicotiana tabacum*

Supplementary Table 3 The predicted cis-element of each *NtBAG* genes
