# Corrigendum: The Bcl-2-associated athanogene gene family in tobacco (*Nicotiana tabacum*) and the function of *NtBAG5* in leaf senescence

**DOI:** 10.3389/fpls.2023.1186777

**Published:** 2023-03-27

**Authors:** Linxin Gu, Bing Hou, Xiao Chen, Yu Wang, Pingan Chang, Xiaohong He, Daping Gong, Quan Sun

**Affiliations:** ^1^ Chongqing Key Laboratory of Big Data for Bio Intelligence, College of Bioinformation, Chongqing University of Posts and Telecommunications, Nan’an, Chongqing, China; ^2^ Tobacco Research Institute, Chinese Academy of Agricultural Sciences, Qingdao, China

**Keywords:** tobacco, BAG protein, leaf senescence, *Nicotiana tabacum*, Bcl-2-associated athanogene

In the published article, there was an error in the legend for **Figure 5**. The gene name was displayed as “PMEI13” in **Figures 5A, B** legend. The corrected legend appears below.

“**Figure 5** Localization of *NtBAG5c* in epidermal cells of *N. benthamiana.* **(A)** Subcellular localization analysis demonstrated that NtBAG5c is located in the cell membrane and cell wall. **(B)** After the wall separation, subcellular localization analysis indicated that NtBAG5c is located in the cell wall. GFP, green fluorescent protein; DAPI, fluorescent dye capable of binding strongly to DNA; Bright, white light; Merged, superposition of GFP, DAPI, and Bright. **(C, D)** Yeast two-hybrid assay. **(C)** The interaction of NtBAG5c and HSP70 in yeast cells. **(D)** The interaction of NtBAG5c and HSP20 in yeast cells. BD-53 + AD-T and BD + AD were used as positive and negative controls, respectively. The yeast co-transformed groups were grown on the SD Leu-Trp medium [double dropout (DDO), without leucine and tryptophan], and then grown on SD-Leu-Trp-His-Ade medium [quadruple dropout (QDO), with leucine, tryptophan, histidine, and adenine.”

**Figure 5:**
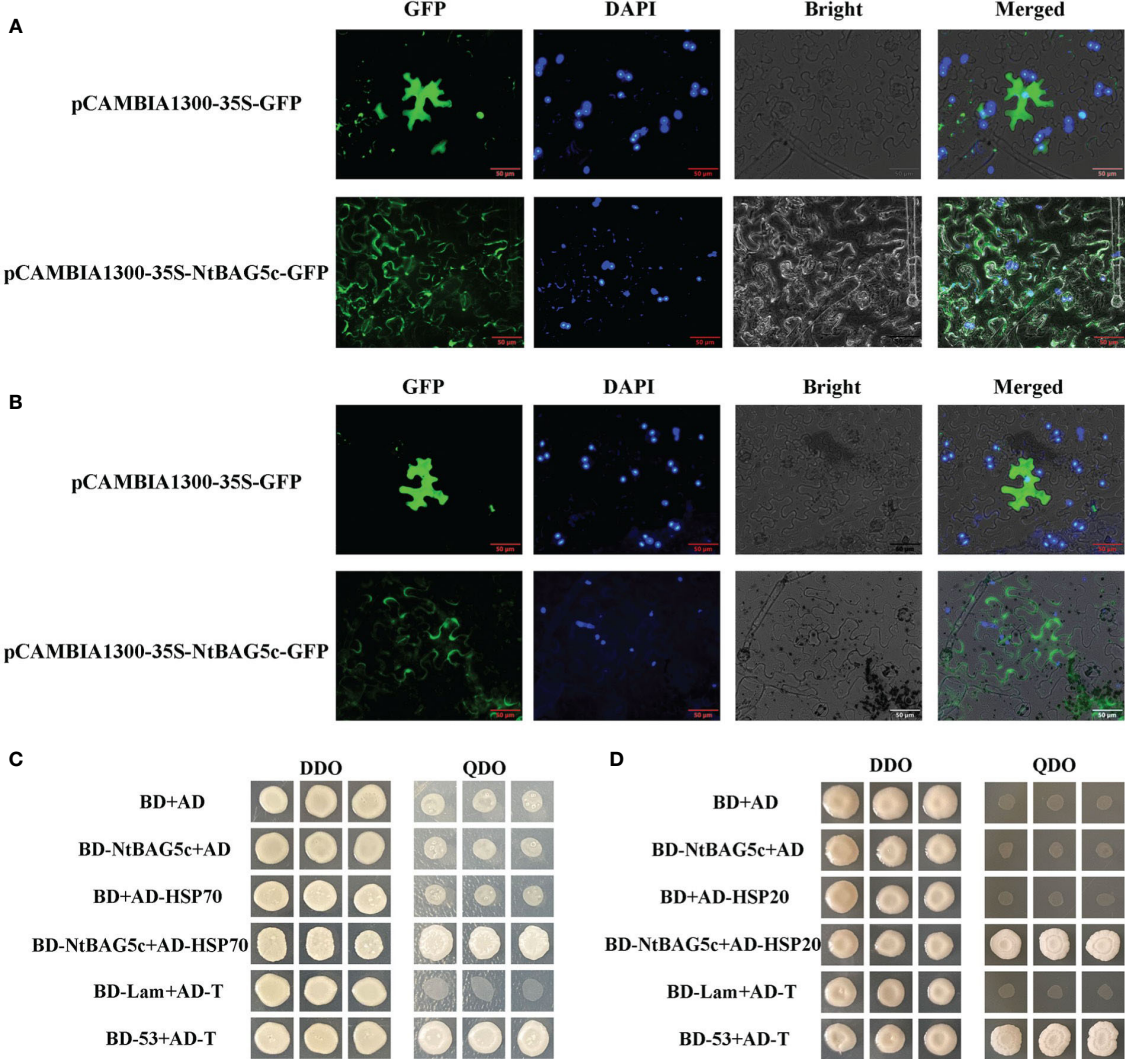
Localization of *NtBAG5c* in epidermal cells of *N. benthamiana*. **(A)** Subcellular localization analysis demonstrated that NtBAG5c is located in the cell membrane and cell wall. **(B)** After the wall separation, subcellular localization analysis indicated that NtBAG5c is located in the cell wall. GFP, green fluorescent protein; DAPI, fluorescent dye capable of binding strongly to DNA; Bright, white light; Merged, superposition of GFP, DAPI, and Bright. **(C, D)** Yeast two-hybrid assay. **(C)** The interaction of NtBAG5c and HSP70 in yeast cells. **(D)** The interaction of NtBAG5c and HSP20 in yeast cells. BD-53 + AD-T and BD + AD were used as positive and negative controls, respectively. The yeast co-transformed groups were grown on the SD Leu-Trp medium [double dropout (DDO), without leucine and tryptophan], and then grown on SD-Leu-Trp-His-Ade medium [quadruple dropout (QDO), with leucine, tryptophan, histidine, and adenine].

The authors apologize for this error and state that this does not change the scientific conclusions of the article in any way. The original article has been updated.

