# Supplementary figures and images for: The Bcl-2-associated athanogene gene family in tobacco (Nicotiana tabacum) and the function of NtBAG5 in leaf senescence
Source: Front Plant Sci. 2023 Feb 9;14:1108588. doi: 10.3389/fpls.2023.1108588 (PMC9947661; doi:10.3389/fpls.2023.1108588)

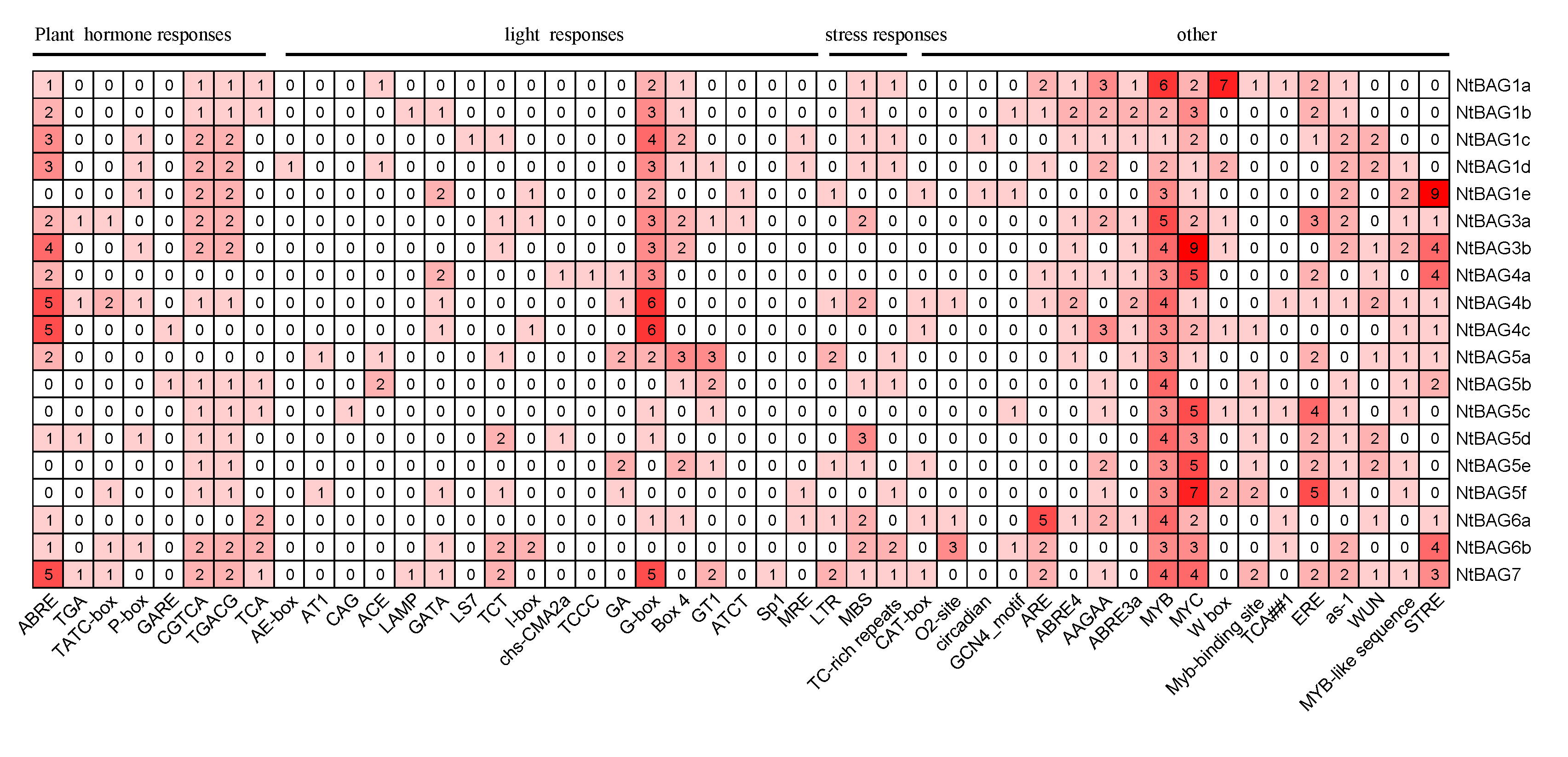

Supplement: Supplementary Figure 1 — Cis-acting elements on promoters (1500 bp) of NtBAG genes. The number shows the number of cis-acting elements. [file Image_1.tiff]
